# Plasma Fibrin Clot Properties as Determinants of Bleeding Time in Human Subjects: Association with Histidine-Rich Glycoprotein

**DOI:** 10.1155/2020/7190828

**Published:** 2020-01-25

**Authors:** Konstanty Szułdrzyński, Miłosz Jankowski, Daniel P. Potaczek, Anetta Undas

**Affiliations:** ^1^Department of Interdisciplinary Intensive Care, Jagiellonian University Medical College, Krakow, Poland; ^2^Department of Anesthesiology and Intensive Care, University Hospital, Krakow, Poland; ^3^Department of Medicine, Jagiellonian University Medical College, Krakow, Poland; ^4^John Paul II Hospital, Kraków, Poland; ^5^Institute of Laboratory Medicine, Member of the German Center for Lung Research (DZL) and Universities of Giessen and Marburg Lung Center (UGMLC), Philipps-University Marburg, Marburg, Germany; ^6^Institute of Cardiology, Jagiellonian University Medical College, Krakow, Poland

## Abstract

**Aims:**

Fibrin formation and histidine-rich glycoprotein (HRG) are involved in primary hemostasis and wound healing. Little is known regarding the relationship of clot characteristics, bleeding time, and wound healing.

**Methods and Results:**

We studied 154 patients with coronary artery disease (CAD) and 154 subjects free of CAD matched for age, obesity, and current smoking. We evaluated bleeding time (BT) using standardized skin incisions on a forearm, along with plasma clot permeability (*K*_s_), clot lysis time (CLT), and histidine-rich glycoprotein (HRG). Compared with controls, BT was 45% shorter in CAD cases. CAD patients had 32% lower *K*_s_), clot lysis time (CLT), and histidine-rich glycoprotein (HRG). Compared with controls, BT was 45% shorter in CAD cases. CAD patients had 32% lower *p* < 0.001). After adjusting for potential confounders, *K*_s_), clot lysis time (CLT), and histidine-rich glycoprotein (HRG). Compared with controls, BT was 45% shorter in CAD cases. CAD patients had 32% lower *n* = 79, 25.6%) was independently predicted by both short and prolonged BT in CAD cases (OR 21.87, 95% CI 7.41-64.55 and OR 10.17, 95% CI 2.88-35.97) and controls (OR 5.94, 95% CI 2.29-15.41 and OR 14.76, 95% CI 4.29-50.77, respectively).

**Conclusions:**

The study shows that plasma fibrin clot density and HRG may influence BT and that appropriate skin wound healing is associated with medium BT. *Translational Perspective*. Elucidation of the complex relationships between plasma fibrin clot phenotype and wound healing might have important practical implications.

## 1. Introduction

Fibrin formation is the final step of blood coagulation involved in hemostasis following vascular injury and the maintenance of vessel patency under pathological conditions. There is evidence that plasma fibrin clots composed of compact networks that are less susceptible to lysis, the so-called “prothrombotic clot phenotype,” can be observed in patients with myocardial infarction (MI), ischemic stroke, and peripheral artery disease [1, 2]. Unfavourable fibrin clot characteristics were also found in patients with cardiovascular risk factors, including cigarette smoking [3], diabetes mellitus [4], and arterial hypertension [5]. The impact of environmental factors is considered prevalent in the prothrombotic plasma clot phenotype [6, 7]. Some medications, in particular aspirin and statins, have been reported to increase clot permeability and susceptibility to lysis [8, 9]. Of importance, recent studies have suggested that clot properties may predict recurrent thrombosis [10].

Fibrin is essential for wound healing as it provides a scaffold for macrophages and fibroblasts migrating into the wound [11]. Fibrin acts together with platelets producing growth factors necessary for recruitment and activation of fibroblasts [12]. Fibrin forms a network stabilizing aggregated platelets. Endothelial cells (ECs) invade the wound soon after leukocytes and monocytes and bind to fibrin with integrins [12]. ECs are activated by vascular endothelial growth factor (VEGF), fibroblast growth factor-2 (FGF-2), fibronectin, and also fibrin while platelet factor-4 (PF4) is one of the most important inhibitors of angiogenesis. Fibrinogen bound to the integrins exposed on the surface of the nucleated cells is necessary to trigger change of cell shape and proper contraction of the wound [12]. The role of abnormal fibrin clot features in defective wound healing has been suggested by clinical phenotypes of some dysfibrinogenaemias [13–15]. Application of a fibrin sealant to the wound accelerates its healing, probably due to improved proliferation and migration of fibroblasts [12]. However, exact relationships between fibrin clot properties assessed in plasma, bleeding time (BT), and the wound healing in human subjects remain unclear. Furthermore, we hypothesized that histidine-rich glycoprotein (HRG) may play role in these relationships. HRG is a liver-produced ~75 kDa single polypeptide chain protein [16] present at ~1.5 *μ*M concentration in human plasma [17]. HRG binds to a diverse range of ligands (fibrin, fibrinogen, plasminogen, heparin, heparan sulfate, thrombospondin, complement C1q, immunoglobulin G, heme, zinc ions, phospholipids, etc.). This may imply its regulatory role for immune, vascular, and coagulation systems [17–19].

Standardized skin incisions performed with a single-use device (e.g., SimPlate or Surgicutt) have been long employed to measure BT in patients suspected of bleeding disorders [20, 21]. Blood coagulation initiated by skin incisions depends on the tissue factor- (TF-) mediated extrinsic pathway with massive thrombin generation in blood collected at the site of injury [22]. Prolonged BT has been shown in, i.e., hemophilia or afibrinogenemia [23, 24]. Interestingly, it has been shown that fibrin formation occurs as early as 30-60 seconds after the skin incision in healthy humans [25]. It is however unclear whether fibrin clot characteristics affect BT in subjects free of bleeding tendency.

To our knowledge, there have been no studies exploring plasma clot features in relation to BT and skin wound healing in healthy subjects and patients with coronary artery disease (CAD) known to form denser fibrin clot structures [6, 7, 9]. We hypothesized that looser fibrin fibre networks prolong BT and impair skin wound healing. We sought to investigate whether plasma clot properties affect BT and scar formation at the site of standardized skin incisions in humans.

## 2. Materials and Methods

### 2.1. Patients

We assessed 154 consecutive patients (cases) with stable CAD (Canadian Cardiovascular Society classes II or III) and, as controls, 154 unrelated subjects free of CAD, who were recruited from the hospital personnel or family members. All study participants were enrolled between 1999 and 2007 and were partly described previously [9, 22]. Patients were excluded if they had any acute illness, known cancer, chronic inflammatory disease (C − reactive protein [CRP] > 10 mg/L), liver injury (alanine transaminase > 1.5 times above the upper limit of the reference range), glomerular filtration rate (GFR) < 30 mL/min, prior venous thromboembolism or stroke, any acute coronary syndrome (ACS) within the 6 preceding months, and current antithrombotic treatment other than acetylsalicylic acid (ASA). The subjects with poor skin condition including scars, edema, atrophy (also all treated with corticosteroids) were ineligible.

Obesity was defined as body mass index (BMI) ≥ 30 kg/m^2^. Diabetes was defined as a history of diabetes, the use of hypoglycemic agents, or fasting plasma glucose ≥126 mg/dL (7 mM) on two separate occasions. Arterial hypertension was defined as a systolic and/or a diastolic blood pressure ≥ 140 mmHg or ≥90 mmHg, respectively, or antihypertensive therapy. Heart failure was defined according to the European guidelines [26]. Anemia was defined as hemoglobin levels < 120 g/L.

The study was performed in accordance with the Declaration of Helsinki and its protocol was approved by the Bioethical Committee of the Jagiellonian University. All subjects enrolled provided written informed consent.

### 2.2. Laboratory Investigations

Fasting blood was drawn from an antecubital vein with minimal stasis between 8 and11 AM. Lipid profiles, blood cell counts, glucose, creatinine, D-dimer, international normalized ratio (INR), and activated partial thromboplastin time (APTT) were assayed by routine laboratory techniques. Factor VIII activity (FVIII) was measured by one-stage clotting assay using factor-deficient plasma (Siemens, Marburg, Germany). Fibrinogen was determined using the von Clauss method. High-sensitivity CRP was measured by nephelometry (Siemens, Marburg, Germany). Fasting plasma tHcy levels were measured using HPLC. Histidine-rich glycoprotein (HRG) and VEGF levels were measured using ELISAs (R&D Systems Inc., Abington, UK). The two parameters were assessed in once thawed samples frozen at -80 degrees Celsius. The interassay and intra-assay coefficients of variation for the ELISAs were <8%.

### 2.3. Bleeding Time

BT assessment was performed by 2 experienced investigators as described previously [27]. In brief, after compressing the upper arm with a sphygmomanometer cuff to 40 mm Hg, two incisions were made on the apparently healthy skin of the lateral aspect of a forearm parallel to the antecubital crease using a Simplate II device (Organon Teknika, Durham, NC, USA). The blood shed from incisions was collected at 30-second intervals and BT was recorded when blood flow ceased in two wounds.

### 2.4. Fibrin Clot Properties

Plasma samples (9 : 1 of 3.2% trisodium citrate) for fibrin assays were centrifuged (20 min, 2500 g) within 30 minutes of collection to obtain platelet-poor plasma, immediately frozen, and stored in aliquots at -80°C. All measurements were performed by technicians blinded to the sample status in the material stored at -80 degrees Celsius and thawed prior to analysis.

#### 2.4.1. Clot Permeability

Permeation of plasma fibrin clots was determined as described [9]. Briefly, 20 mM calcium chloride and 1 U/mL human thrombin (Sigma-Aldrich/Merck, Darmstadt, Germany) were added to citrated plasma. Tubes containing the clots were connected to a reservoir of a Tris-buffered saline (TBS) buffer (0.01 M Tris, 0.1 M NaCl, pH 7.5), and its volume flowing through the gels was measured within 60 minutes. A permeation coefficient (*K*_s_), indicating the pore size, was calculated from the equation: *K*_s_ = *Q* × *L* × *η*/*t* × *A* × Δ*p*, where *Q*is the flow rate in time *t*, *L* is the length of a fibrin gel, *η* is the viscosity of liquid (in poise), *t* is percolating time, *A* is the cross-sectional area (in cm^2^), and Δ*p* is a differential pressure (in dyne/cm^2^). The intraindividual variability of results was 8%.

#### 2.4.2. Clot Lysis Time

CLT was measured as described [28, 29]. Briefly, citrated plasma was mixed with 15 mM calcium chloride, 10,000-diluted human TF (Innovin, Siemens, Marburg, Germany), 12 *μ*M phospholipid vesicles (highly purified phosphatidylcholine, phosphatidylserine, and sphingomyelin from Rossix, Molndal, Sweden), and 60 ng/ml recombinant t-PA (Boehringer Ingelheim, Ingelheim, Germany). The mixture was transferred to a microtiter plate and its turbidity was measured at 405 nm at 37°C. Clot lysis time was defined as the time from the midpoint of the clear-to-maximum-turbid transition, which represents clot formation, to the midpoint of the maximum-turbid-to-clear transition. The coefficients of intra- and interassay variations were 6%.

### 2.5. Scar Formation

Formation of the scar at the site of prior BT test was assessed macroscopically by the independent investigators two weeks after BT measurement. The scar was defined as a linear mark at the site of skin incision different in colour from the surrounding skin and >1 mm wide. The interobserver variability was estimated at 10%, based on evaluation of 50 randomly selected patients performed independently by 2 investigators.

All methods were performed in accordance with the relevant guidelines and regulations.

### 2.6. Statistical Analysis

The study was powered to have a 90% chance of detecting a 10% difference in *K*_s_ using a *p* value of 0.05. To demonstrate such a difference or greater in *K*_s_, at least 31 patients were required in each group [9].

Categorical variables were compared using *χ*^2^ exact test. Continuous variables were tested for the normality of the distribution by the Shapiro-Wilk *W* test. Those normally distributed are given as the mean ± standard deviation and otherwise as median (interquartile range). Comparisons were performed using unpaired (Student's) *t*-test or Mann–Whitney *U* test, respectively. Associations between continuous variables were calculated using Spearman's rank correlation coefficient. Uni- and multivariate logistic regression models were used to determine predictors of scar formation. We determined predictors of long and short BT, defined as the upper and the lower BT quartile, respectively, along with predictors of a very long BT with its 90^th^ percentile as a cutoff value. Multivariate analyses adjusted for age, diabetes, ASA use, and statin treatment. Models for BT prediction involved also current smoking, platelet count, FVIII, CLT, *K*_s_, and HRG. A *p* value of less than 0.05 was considered significant. Statistical analyses were conducted using the Statistica 64 v.13 (StatSoft/Dell, Tulsa, OK, USA) or MedCalc 64-bit v.13.1.2.0 (MedCalc Software, Ostend, Belgium) program.

## 3. Results

### 3.1. Characteristics of the Studied Groups

The CAD group had a lower proportion of females and a higher prevalence of hypertension, diabetes, higher BMI, total cholesterol, and LDL cholesterol as well as a more frequent statin use, compared with controls (Table 1). Fibrinogen and FVIII levels were higher in CAD patients than controls (Table 1).

### 3.2. Bleeding Time

BT was 45% shorter in CAD patients than controls (Table 1). An inverse correlation between BT and age was observed only in controls (*R* = −0.54, *p* < 0.001). As expected, BT was longer in CAD patients treated by ASA (250 [211-309] vs. 229 [199-275] sec, respectively, *p* = 0.03), while those on statins had shorter BT (210 [195-250] vs. 240 [207-296] sec, respectively, *p* = 0.01).

In the CAD and control groups, there were inverse correlations between BT and fibrinogen (*R* = −0.30, *p* < 0.001 and *R* = −0.52, *p* < 0.001, respectively) and platelet count (*R* = −0.51, *p* < 0.001 and *R* = −0.45, *p* < 0.001, respectively). There was an inverse weak correlation between BT and FVIII (*R* = −0.21, *p* = 0.01) in controls who all had FVIII above 80%.

### 3.3. HRG and VEGF

Plasma HRG levels were 50% lower in CAD patients than in controls (Table 1). HRG inversely correlated with age (*R* = −0.27, *p* < 0.001) and fibrinogen (*R* = −0.24, *p* = 0.003) in controls. In both CAD patients and controls, HRG correlated negatively with platelet count (*R* = −0.25, *p* = 0.001 and *R* = −0.21, *p* = 0.008, respectively) and CRP (*R* = −0.16, *p* = 0.04 and *R* = −0.26, *p* = 0.001, respectively). Interestingly, there was a positive correlation of HRG with BT (*R* = 0.39, *p* < 0.001 and *R* = 0.43, *p* < 0.001, respectively) in both groups. There was no difference in HRG and VEGF between subjects whose plasma samples were collected within the first 2 years and those from the remaining years (data not shown).

VEGF levels were 147% higher in CAD patients compared with controls (Table 1). VEGF correlated positively with age (*R* = 0.44, *p* < 0.001) and fibrinogen (*R* = 0.26, *p* < 0.001) only in controls. We observed inverse correlation between VEGF and BT (*R* = −0.25, *p* = 0.002) in controls, but not in CAD patients.

### 3.4. Fibrin Clot Properties

**Figure 1:**
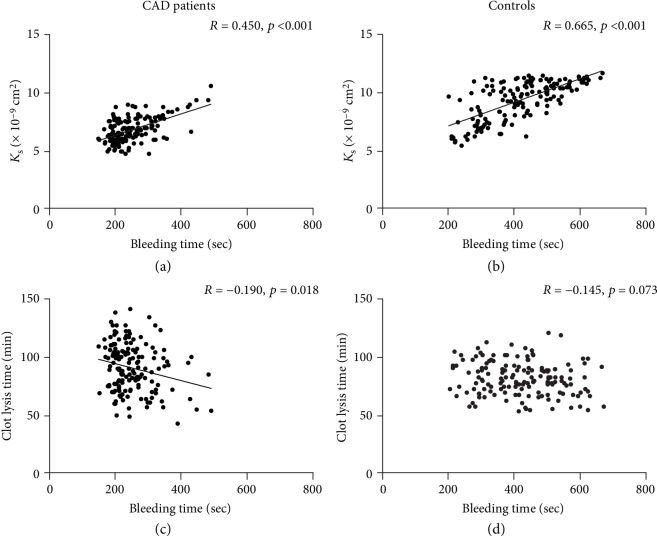
Correlations between bleeding time and fibrin clot permeability (*K*_s_ (a, b)) and lysis time (c, d) in CAD patients (*n* = 154; a, c) and controls (*n* = 154; b, d). *p* values calculated using Spearman's rank correlation coefficient.

**Figure 2:**
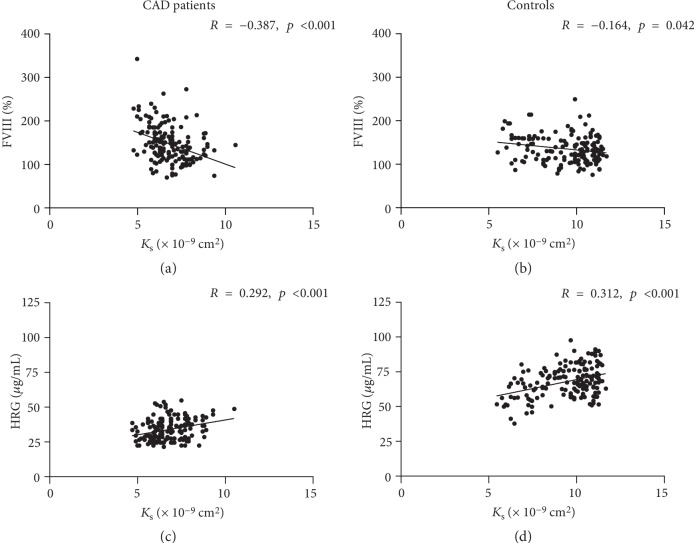
Correlations between fibrin clot permeability (*K*_s_) and FVIII (a, b) and HRG (c, d) in CAD patients (*n* = 154; a, c) and controls (*n* = 154; b, d). *p* values calculated using Spearman's rank correlation coefficient.

As expected, plasma fibrin clots were denser (32% lower *K*_s_) and more resistant to fibrinolysis (17% longer CLT) in CAD patients compared with controls (Table 1). Comorbidities showed no associations with clot properties. Among medications, only ASA use was associated with higher *K*_s_ in CAD patients (7.0 [6.3 − 8.0] vs.6.5 [6.0 − 7.4] × 10^−9^ cm^2^, *p* = 0.01). Of note, positive correlations between *K*_s_ and BT were observed in both CAD (Figure 1(a)) and control (Figure 1(b)) groups. In CAD patients (Figure 1(c)), but not in controls (Figure 1(d)), there was also a weak inverse correlation between CLT and BT. In both CAD patients and controls, there were inverse correlations between *K*_s_ and FVIII (Figures 2(a) and 2(b)), and positive correlations between *K*_s_ and HRG (Figures 2(c) and 2(d)). VEGF inversely correlated with *K*_s_ (*R* = −0.28, *p* < 0.001) in controls but not in CAD patients. HRG or VEGF showed no associations with CLT in either group. The duration of storage at -80 degrees Celsius showed association with neither *K*_s_ nor CLT (data not shown).

### 3.5. Determinants of BT

In CAD patients, univariate analysis showed that diabetes and ASA treatment were associated with higher chance of very long BT values, i.e., above the 90^th^ percentile (≥336 sec; Table 2). Less-dense fibrin clot formation, defined as *K*_s_ values in the top quartile, was a predictor of both a long (≥279 sec, the upper quartile, Supplementary [Supplementary-material supplementary-material-1]) and a very long BT (Table 2). HRG levels in the upper quartile (but not CLT) were also associated with long and very long BT (Supplementary [Supplementary-material supplementary-material-1], Table 2). Statin use, elevated platelet count (the upper quartile), and FVIII > 150% were in turn inversely related to long BT (Supplementary [Supplementary-material supplementary-material-1]). Multivariate analyses showed that *K*_s_ had its predictive value for both a long and a very long BT; high HRG levels were significantly associated only with a very long BT and platelet count exclusively with a long BT (Table 2 and Supplementary [Supplementary-material supplementary-material-1]).

In controls, age was associated with lower chance of both long and very long BT (Supplementary [Supplementary-material supplementary-material-1] and Table 2). Platelet count in the top quartile was inversely associated with BT above the upper quartile (Supplementary [Supplementary-material supplementary-material-1]). Moreover, both *K*_s_ and HRG correlated positively with long and very long BT, but CLT was not associated with BT (Supplementary [Supplementary-material supplementary-material-1] and Table 2). In the multivariate model for prediction of a very long BT including age, current smoking, anemia, high platelet count, FVIII above 150%, high CLT, high *K*_s_, and high HRG, the effects of the latter two remained significant (Table 2). In a similar multivariate model with a long BT as the dependent variable, the effect of age, platelet count, and HRG, but not that of *K*_s_, remained significant (Supplementary [Supplementary-material supplementary-material-1]).

In both univariate and multivariate analyses, short BT (i.e., below the lower quartile) was associated in CAD patients with statin treatment as well as platelets, *K*_s_, and HRG below their lower quartiles (Table 3). In controls in turn, short BT was predicted by age, low *K*_s_, and low HRG in univariate analysis but only the effect of *K*_s_ remained significant in the multivariate model (Table 3).

### 3.6. Scar Formation

Scar formation at sites of BT measurement was observed in 41 (26.6%) CAD patients and 38 (24.7%) controls. CAD patients with scars were more frequently affected by diabetes and treated with ASA or statins and had shorter BT (by 18%) compared with the remainder (Supplementary [Supplementary-material supplementary-material-1]). Control subjects with scars were older, less frequently females and smokers compared to the remainder. Moreover, 12% lower *K*_s_ together with 36% higher homocysteine and 41% higher CRP (all *p* < 0.05) were observed in controls who developed scars following skin incisions (Supplementary [Supplementary-material supplementary-material-1]).

**Figure 3:**
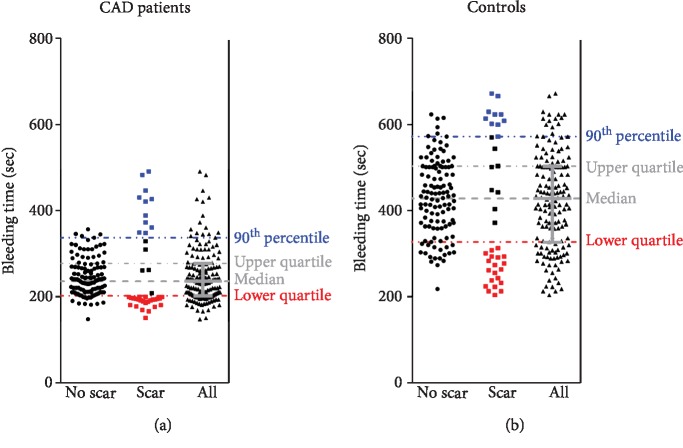
Distribution of bleeding time (BT) values in CAD patients (*n* = 154; a) and controls (*n* = 154; b) with and without scars following BT measurement.

Analysis of association between BT and scar formation demonstrated that scars developed almost exclusively (in CAD patients) or mostly (controls) in subjects with a short (in the lower quartile) or a very long (above 90^th^ percentile) BT (Figure 3). Not surprisingly, univariate analyses of scar formation predictors showed that both CAD patients with diabetes and treated with ASA or statin, and with a short or a very long BT, had an increased risk of scar formation after skin incisions (Table 4). Multivariate models were developed to assess the effect of age, diabetes, and ASA or statin treatment on scar formation, and either a short or a very long BT. In the first model, the effects of a short BT, diabetes, and statin treatment remained significant. In the other, a very long BT, statin treatment, and age were predictors of scar formation. In control subjects, both a low and a very long BT and, additionally, age were associated with an increased risk of scars following skin incisions in univariate analyses. Those effects remained significant in multivariate analyses, except for that observed for age in the model involving a short BT (Table 4). A long BT (the top quartile) was associated with impaired wound healing only in the control group (Supplementary [Supplementary-material supplementary-material-1]).

## 4. Discussion

To our knowledge, this study is the first to show that increased permeability of plasma fibrin clots is correlated with longer BT in human subjects. We demonstrated that *K*_s_ is an independent predictor of longer BT in both studied groups suggesting that fibrin network density affects bleeding time and has the impact on the primary hemostasis. Another novel finding is that elevated HRG in circulating blood correlates with longer BT in humans, which suggests that this abundant protein may affect primary hemostasis at least in part through its impact on fibrin clot structure. We showed an interesting U-shaped relationship between BT and scar formation following skin incisions, suggesting that appropriate skin wound healing is associated with medium BT. The current study expands our knowledge on the role of fibrin clot properties in primary hemostasis and wound healing in healthy subjects and cardiovascular patients, independently of fibrinogen and platelet count.

Previously, it has been shown that plasma fibrin clot characteristics may influence bleeding risk as observed in women with heavy menstrual bleeding [29] and patients with atrial fibrillation receiving vitamin K antagonists [30]. To our knowledge, there have been no published reports suggesting that mucocutaneous bleeding or bleeding from superficial skin injuries in healthy subjects may be linked to fibrin clot properties determined in plasma-based assays. The present study shows that apart from platelet count, the density of plasma fibrin clots could contribute to BT independently of fibrinogen concentrations, suggesting a significant involvement of fibrin in primary hemostasis in health and prothrombotic states with their commonest representative, CAD. Recently, Macrae et al. [31] have described a thin fibrin biofilm covering a blood clot at the contact with air. Whether this phenomenon might contribute to shorter BT in subjects with denser clot network formation which could facilitate formation of fibrin biofilm following skin injury is worth investigating. Interesting relationships between the mass of the clot fibrin matrix and the balance of pro- and antiangiogenic factors were shown by Hadjipanayi et al. [32]. Increasing the clot mass shifted the balance towards proangiogenic VEGF instead of antiangiogenic PF4. Also, clot hypoxia promoted proangiogenic factors. Moreover, EC migration into the fibrin matrix was enhanced proportionally to increasing mass of the clot matrix. These observations suggest that the matrix controls the process of transition from hemostasis to angiogenesis by regulation of pro- and antiangiogenic balance. However, these observations provide little insight into the relationship between the characteristics of the fibrin clot arising from its spatial structure and the ability to heal the wound properly.

In our study, BT was shorter in CAD patients compared to controls, which corroborates with earlier findings [33], but also in subjects who formed dense fibrin clots based on the plasma-based assays. Fibrin is formed within tens of seconds from vascular injury [25], but the stability and function of the resultant fibrin clot depends on its spatial structure impacting the properties of the thrombus at the site of injury. The relationship between clot susceptibility to lysis and BT was weak and detectable only in CAD patients, suggesting a negligible impact of clot lysability measured *in vitro* on BT.

The current study is the first to show that plasma levels of HRG are involved in primary hemostasis in both CAD patients and controls. We found that HRG correlated positively with BT, which provides evidence for a role of this anticoagulant and antifibrinolytic protein *in vivo* in humans. HRG interacts with fibrinogen while assessed *in vitro* which leads to the formation of less compact fibrin clots [18]. In animal models, HRG deficiency has been reported to enhance thrombin generation and promote arterial thrombosis [34]. It is estimated that nearly 50% of plasminogen circulates bound to HRG, which reduces plasminogen available to bind fibrin [19, 35]. Moreover, HRG can be incorporated into fibrin clots and its presence causes the formation of thinner fibrils as demonstrated in a purified system. Vu et al. [36] demonstrated that HRG competes with thrombin for binding site of *γ*′-fibrinogen, known to increase fibrin clot density [37]. On the other hand, Kotzé et al. [38] have shown that fibrin clot properties are related to cardiovascular risk factors independently of *γ*′-fibrinogen levels. The present study demonstrated that higher HRG levels are associated with looser plasma clot networks in human subjects, suggesting additional anticoagulant effects of HRG reported *in vitro* [18, 19, 34, 35]. The study performed in HRG-deficient mice showed higher spontaneous fibrinolytic activity but shorter prothrombin and bleeding time in the absence of HRG compared to controls [39]. The present study failed to show any associations between HRG and CLT; however, we cannot exclude that other assays used to assess fibrinolysis might show antifibrinolytic effects of this protein suggested by *in vitro* experiments. Of note, the presence of HRG in plasma clots in patients with deep vein thrombosis has been shown recently [40]. Taken together, the current findings suggest that HRG is involved in human hemostasis and may modulate fibrin clot structure *in vivo*.

Unexpectedly, a very long as well as a short BT correlated with skin scar formation. We hypothesized that more compact and less permeable clots would be associated with impaired wound healing due to attenuated fibroblast invasion into the wound space filled with dense fibrin network [12]. As longer BT was associated with less compact and more permeable fibrin network, it may be presumed that longer bleeding impairs formation of the provisional scaffold necessary for successful wound healing [12, 41]. We failed to observe association between fibrin clot properties and scar formation. It might suggest that the structure of fibrin clots in a range encountered in most human subjects (without extremes) is sufficiently “loose” to ensure appropriate wound healing, with no major impact on formation of skin scars or not. A strong negative correlation between scar formation and platelet count in the control group supports the role of platelets not only in the primary hemostasis but also in proper clot contraction and secretion of growth factors indispensable for appropriate healing. We did not show the same correlation in the CAD group, likely due to more frequent ASA use. Therefore, an intriguing finding that scar formation is associated with a short or a very long BT is hard to explain. It may be hypothesized that scar formation is associated with various processes depending on whether a subject has either a short or a very long BT. It is also possible that proper wound healing requires the good quality of the skin and its vessels along with optimal blood coagulation, reflected at least in part by an “average” BT and all alterations lead to defective tissue repair facilitating scar formation. We also noted that CAD patients with scars were more frequently diabetic and treated with ASA or statins than those without scars. Diabetes is known to increase density of fibrin clots, while both aspirin and statins have been reported to render clot networks looser, though not in diabetic patients [9, 42]. Our observations indicate that skin scar formation is influenced by comorbidities and medications used with a particular impact of diabetes. Since the ability of proper wound healing depends on multiple environmental or acquired variables [43], we identified new potential modulators of this complex process. Further research is needed to elucidate regulation of skin scar formation and its potential practical implications, e.g., for treatment of bleeding, surgical and trauma wounds, chronic skin ulcers, bedsores, and in plastic surgery.

Interestingly, we observed inverse correlations between VEGF and BT as well as between VEGF and *K*_s_ in controls, but not in CAD patients. Most likely, these associations reflect the impact of endothelial damage, along with platelets as an important source of circulating VEGF, a major growth factor stimulating angiogenesis during wound healing [44, 45]. Despite higher circulating VEGF levels in the CAD group, we did not observe any correlation between VEGF and scar formation, which might have been expected as a result of impaired neovascularization [43]. This issue requires further studies.

The current study has several limitations. The number of subjects in the study was limited; however, the study was adequately powered to detect significant differences in fibrin clot characteristics. It should be noted that the observed associations do not necessarily mean the cause-effect relationship. Several other factors that may modulate clot properties have not been assessed, for example, complement C3, fibronectin, and some genetic determinants [6, 7]. We also did not measure levels of *γ*′-fibrinogen, which is known to alter clot structure [11, 46], which could be one of additional contributors to the phenomena described here; however, given prevalence of this variant, it is unlikely that *γ*′-fibrinogen explains all differences and associations observed by us. The same holds true for factor XIII levels, a potent modulator of fibrin clot structure [47]. Moreover, we did not measure von Willebrand factor, which may influence clot structure and BT. Furthermore, to better characterize fibrin clot properties, scanning electron microscopy of clots and thromboelastography could be used in future studies. The use of other techniques including the PFA-100 and ROTEM would be helpful in assessment of the association between fibrin clot properties and bleeding; however, such analyses were beyond the scope of this hypothesis-generating study and will be applied in future studies. Finally, *in vitro* experiments, especially elucidating the impact of HRG and VEGF on clots, are needed to get more mechanistic insights into the phenomena presented; however, this study is hypothesis-generating and strongly supports further research in this field given observations made in patients and control subjects.

To conclude, this study indicates that fibrin clot structure may affect BT in humans, suggesting a role of fibrin properties in primary hemostasis. The current study demonstrated a potential role of circulating HRG in primary hemostasis and its possible impact on fibrin clot structural properties. Further studies are warranted to elucidate complex relationships between plasma fibrin clot phenotype and wound healing, which might have important practical implications.

## Figures and Tables

**Table 1:** Characteristics of study groups.

	CAD patients (*n* = 154)	Controls (*n* = 154)	*p*
Age (y)	64 (59-70)	61 (58-68)	0.05
Females, *n* (%)	74 (48)	93 (60)	0.03
BMI (kg/m^2^)	28 (24-31)	24.9 (22.6-27.6)	<0.001
Risk factors of CAD			
Current smoking, *n* (%)	53 (34)	52 (34)	0.90
Arterial hypertension, *n* (%)	90 (58)	51 (33)	<0.001
Diabetes mellitus, *n* (%)	24 (15.6)	—	—
Prior MI, *n* (%)	42 (27.7)	—	—
Treatment, *n* (%)			
ASA	43 (28)	—	—
Statin	38 (25)	—	—
*β*-Blocker	39 (25)	—	—
ACEI	63 (41)	—	—
Laboratory investigations			
Hemoglobin (g/dL)	14.07 ± 1.42	13.85 ± 1.31	0.16
Platelet count (× 10^3^/*μ*L)	241 (205-308)	238 (205-302)	0.75
Total cholesterol (mM)	5.16 (4.50-5.84)	4.88 (4.27-5.57)	0.04
LDL cholesterol (mM)	3.30 (2.75-3.95)	3.02 (2.46-3.69)	0.005
Glucose (mM)	5.3 (5.0-5.8)	5.3 (4.9-5.7)	0.31
Homocysteine (*μ*M)	12.2 (10.1-19.1)	11.1 (10.0-15.7)	0.11
INR	1.00 (0.97-1.06)	1.00 (0.97-1.05)	0.75
APTT (sec)	27.1 (24.7-30.0)	27.2 (25.0-30.4)	0.69
FVIII (%)	142 (117-173)	131.5 (111-158)	0.009
Fibrinogen (g/L)	3.36 (2.80-4.00)	2.79 (2.37-3.22)	<0.001
CRP (mg/L)	1.93 (1.40-2.58)	1.69 (1.28-2.39)	0.06
BT (sec)	235 (202-277)	424 (324-500)	<0.001
CLT (min)	90 (74-106)	77 (68-88)	<0.001
*K*_s_ × 10^−9^ cm^2^	6.7 (6.1-7.6)	9.8 (8.0-10.7)	<0.001
VEGF (pg/mL)	72.4 (49.8-88.0)	29.3 (25.6-34.9)	<0.001
HRG (*μ*g/mL)	33.0 (28.0-40.0)	65.9 (57.2-74.2)	<0.001

CAD: coronary artery disease; BMI: body mass index; MI: myocardial infarction; ASA: acetylsalicylic acid; ACEI: angiotensin-converting enzyme inhibitor; LDL: low-density lipoprotein; INR: international normalized ratio; APTT: activated partial thromboplastin time; FVIII: coagulation factor VIII; CRP: C-reactive protein; BT: bleeding time; CLT: clot lysis time; *K*_s_: clot permeability; VEGF: vascular endothelial growth factor; HRG: histidine-rich glycoprotein. Continuous variables are expressed as the mean ± standard deviation or median (interquartile range), as appropriate.

**Table 2:** Determinants of a very long bleeding time (BT, above the 90^th^ percentile) in coronary artery disease (CAD) patients and controls.

Variable	CAD patients (BT ≥ 336 sec)				Controls (BT ≥ 568 sec)			
	Univariate analysis		Multivariate analysis		Univariate analysis		Multivariate analysis	
	OR (95% CI)	*p*	OR (95% CI)	*p*	OR (95% CI)	*p*	OR (95% CI)	*p*
Age (y)	1.01 (0.94-1.09)	0.75	1.01 (0.89-1.13)	0.95	0.77 (0.66-0.90)	<0.001	0.83 (0.69-1.01)	0.06
Current smoking	0.61 (0.19-1.98)	0.41	1.07 (0.20-5.79)	0.94	1.08 (0.38-3.10)	0.89	1.56 (0.37-6.49)	0.54
Diabetes	4.00 (1.30-12.34)	0.016	1.70 (0.24-12.00)	0.59	—		—	
Anemia	2.09 (0.61-7.15)	0.24	1.70 (0.16-17.78)	0.66	0.51 (0.06-4.11)	0.53	1.49 (0.13-16.36)	0.76
ASA treatment	2.94 (1.03-8.43)	0.044	1.84 (0.37-9.04)	0.45	—		—	
Statin treatment	0.18 (0.02-1.43)	0.10	0.18 (0.01-2.24)	0.18	—		—	
Platelet count in the top quartile (≥308 and ≥302 × 10^3^/*μ*L)^∗^	0.65 (0.18-2.43)	0.53	1.59 (0.20-12.47)	0.66	0.16 (0.02-1.27)	0.08	0.18 (0.02-1.81)	0.15
FVIII > 150%	0.78 (0.27-2.27)	0.65	2.51 (0.47-13.22)	0.28	0.50 (0.14-1.84)	0.30	1.62 (0.31-8.50)	0.57
CLT in the top quartile (>106 and >88 min)^∗^	0.39 (0.08-1.80)	0.23	0.71 (0.10-5.11)	0.73	0.97 (0.30-3.18)	0.96	0.73 (0.141-3.85)	0.72
*K*_s_ in the top quartile (>7.6 and ≥10.7 × 10^−9^ cm^2^)^∗^	20.58 (5.44-77.85)	<0.001	23.70 (4.65-120.8)	<0.001	19.92 (5.33-74.44)	<0.001	10.89 (2.31-51.44)	0.003
HRG in the top quartile (≥40 and ≥74.2 *μ*g/mL)^∗^	11.79 (3.53-39.33)	<0.001	10.27 (2.05-51.31)	0.005	4.01 (1.42-11.29)	0.009	4.54 (1.07-19.27)	0.040

OR: odds ratio; CI: confidence interval; otherwise, see Table 1. ^∗^Cutoffs for CAD patients and controls, respectively, are given.

**Table 3:** Determinants of a short bleeding time (BT, the lower quartile) in coronary artery disease (CAD) patients and controls.

Variable	CAD patients (BT < 202 sec)				Controls (BT < 324 sec)			
	Univariate analysis		Multivariate analysis		Univariate analysis		Multivariate analysis	
	OR (95% CI)	*p*	OR (95% CI)	*p*	OR (95% CI)	*p*	OR (95% CI)	*p*
Age (y)	1.02 (0.97-1.08)	0.41	1.01 (0.95-1.08)	0.69	1.20 (1.12-1.28)	<0.001	1.10 (1.00-1.21)	0.06
Current smoking	1.34 (0.63-2.86)	0.45	1.50 (0.57-3.99)	0.42	0.53 (0.23-1.22)	0.13	0.54 (0.18-1.62)	0.27
Diabetes	0.77 (0.27-2.24)	0.64	1.24 (0.31-4.87)	0.76	—		—	
Anemia	1.41 (0.53-3.74)	0.49	1.11 (0.30-4.07)	0.87	1.45 (0.47-4.46)	0.52	1.61 (0.37-7.10)	0.53
ASA treatment	0.62 (0.26-1.48)	0.28	0.42 (0.12-1.44)	0.17	—		—	
Statin treatment	2.24 (1.01-4.97)	0.048	4.34 (1.44-12.97)	0.01	—		—	
Platelet count in the lower quartile (≤205 and ≤205 × 103/*μ*L)^∗^	0.18 (0.05-0.63)	0.007	0.22 (0.05-0.92)	0.038	0.37 (0.13-1.02)	0.05	0.36 (0.10-1.32)	0.12
FVIII > 150%	1.11 (0.53-2.32)	0.79	0.65 (0.23-1.83)	0.41	1.96 (0.90-4.24)	0.09	0.66 (0.21-2.05)	0.48
CLT in the lower quartile (≤74 and <68 min)^∗^	0.73 (0.30-1.77)	0.49	1.02 (0.33-3.16)	0.97	0.93 (0.39-2.20)	0.87	2.53 (0.77-8.33)	0.13
*K*_s_ in the lower quartile (<6.1 and ≤8 × 10^−9^ cm^2^)^∗^	4.32 (1.94-9.60)	<0.001	3.35 (1.15-9.73)	0.027	21.27 (8.47-53.44)	<0.001	10.02 (2.45-40.97)	0.001
HRG in the lower quartile (≤28 and ≤56.9 *μ*g/mL)^∗^	9.23 (4.04-21.07)	<0.001	9.19 (3.50-24.29)	<0.001	3.11 (1.40-6.87)	0.005	1.11 (0.36-3.41)	0.85

For a list of abbreviations, see Tables 1 and 2. ^∗^Cutoffs for CAD patients and controls, respectively, are given.

**Table 4:** Determinants of scar formation following bleeding time (BT) measurement in coronary artery disease (CAD) patients and controls (including a short and a very long BT).

Variable	CAD patients	*p*	Controls	*p*
	*Univariate analysis*			
Age (y)	1.04 (0.99-1.10)	0.10	1.06 (1.01-1.12)	0.021
Diabetes	3.48 (1.42-8.57)	0.007	—	
ASA treatment	2.37 (1.11-5.07)	0.026	—	
Statin treatment	2.26 (1.03-4.94)	0.042	—	
BT above the 90^th^ percentile (≥336 and ≥568 sec)^∗^	7.92 (2.55-24.56)	<0.001	5.56 (1.94-15.91)	0.001
BT in the lower quartile (<202 and <324 sec)^∗^	12.02 (5.12-28.21)	<0.001	6.05 (2.69-13.62)	<0.001
	*Multivariate model with BT above the 90^th^ percentile*			
Age (y)	1.06 (1.00-1.13)	0.035	1.12 (1.05-1.19)	<0.001
Diabetes	2.65 (0.91-7.68)	0.07	—	
ASA treatment	1.19 (0.47-3.00)	0.72	—	
Statin treatment	3.91 (1.51-10.13)	0.005	—	
BT above the 90^th^ percentile (≥336 and ≥568 sec)^∗^	10.17 (2.88-35.97)	<0.001	14.76 (4.29-50.77)	<0.001
	*Multivariate model with BT below the lower quartile*			
Age (y)	1.05 (0.99-1.12)	0.12	1.00 (0.94-1.07)	0.94
Diabetes	5.10 (1.59-16.30)	0.006	—	
ASA treatment	3.56 (1.19-10.66)	0.024	—	
Statin treatment	1.53 (0.53-4.37)	0.43	—	
BT below the lower quartile (<202 and <324 sec)^∗^	21.87 (7.41-64.55)	<0.001	5.94 (2.29-15.41)	<0.001

For a list of abbreviations and legends, see Tables 1 and 2.

## Data Availability

The data used to support the findings of this study are available from the corresponding author upon request.

## Supplementary Materials

Supplementary Materials Supplementary Table S1: determinants of a long bleeding time (BT, above the upper quartile) in coronary artery disease (CAD) patients and controls. Supplementary Table S2: coronary artery disease (CAD) patients and controls with and without scars after bleeding time (BT) test. Supplementary Table S3: determinants of scar formation following bleeding time (BT) testing in coronary artery disease (CAD) patients and controls (including a long BT). Supplementary Table S4: STROBE Statement—checklist of items that should be included in reports of case-control studies.
